# Determining the Prevalence and Correlates of COVID-19 Booster Vaccine Hesitancy in the Singapore Population Following the Completion of the Primary Vaccination Series

**DOI:** 10.3390/vaccines10071088

**Published:** 2022-07-06

**Authors:** Kevin Y. K. Tan, Alexius S. E. Soh, Brenda W. L. Ong, Mark IC. Chen, Konstadina Griva

**Affiliations:** 1 Population/Global Health, Lee Kong Chian School of Medicine, Nanyang Technological University, Singapore 308232, Singapore; kevin.tanyk@ntu.edu.sg; 2 Infectious Disease Research and Training Office, National Centre for Infectious Diseases, 16 Jln Tan Tock Seng, Singapore 308442, Singapore; alexius_matthias_se_soh@ncid.sg (A.S.E.S.); brenda_ong@ncid.sg (B.W.L.O.); mark_ic_chen@ncid.sg (M.I.C.)

**Keywords:** COVID-19, vaccine hesitancy, booster vaccination, Singapore

## Abstract

In response to declining vaccine-induced immunity and the emergence of new COVID-19 variants, COVID-19 booster vaccination programmes have been widely launched in several high-income countries. However, public response has been slow, and scepticism about these programmes is rising in these settings. This study sought to identify the sociodemographic, emotional, and psychological factors associated with COVID-19 booster vaccine hesitancy in Singapore. Derived from a community cohort, 1005 fully vaccinated adults (62.1% female, mean age = 42.6 years) that had not received their COVID-19 booster shots completed an online survey between October and November 2021 on vaccination beliefs, intentions, and behaviours. Results indicated that despite completing the primary COVID-19 vaccination, 30.5% of those surveyed were hesitant about receiving the booster shot (25.9% unsure; 4.7% refused the booster), and 39.2% perceived more vaccine risks than benefits. Multivariable models indicated that a tertiary education, lower COVID-19 threat perception, lower perceived benefits, higher perceived concerns, a decreased need for booster vaccination, and a lower benefit/concerns differential score were associated with higher odds of booster vaccine hesitancy. Success in the primary vaccination series may not warrant widespread public acceptance for recurrent COVID-19 vaccination doses. In addressing booster vaccine hesitancy as restrictive measures and mandates are lifted, health perceptions relevant or unique to booster vaccine uptake should be considered.

## 1. Introduction

COVID-19 vaccines have established their effectiveness and efficacy against severe disease [1,2], but a rapid waning in vaccine-induced immunity over time [3,4], and the emergence of new variants have led to the launch of booster vaccination programmes in several countries. Singapore achieved high COVID-19 vaccination coverage among the eligible population and promptly introduced its vaccination booster programme on 14 September 2021 [5], which at launch included two approved mRNA COVID-19 vaccines for booster vaccination: the Moderna/Spikevax and the Pfizer-BioNTech/Comirnaty vaccine [3]. At the launch of the programme, 81% of the local population had completed the primary series of COVID-19 vaccinations, and no incentives were offered for booster vaccine uptake [6]. The programme was first made available to senior citizens above 60 years old [7], and was subsequently extended to all eligible age groups (individuals 18 years and above) who had completed their primary COVID-19 vaccination regimen at least 5 months prior [8]. As of 13 May 2022, the Novavax/Nuvaxovid vaccine was included in the booster programme and was only made available to individuals 18 years old and above [9]. Despite the wide accessibility and availability of COVID-19 booster vaccines, public response to booster vaccination remained slow until the introduction of booster vaccine mandates in early 2022, which required a booster dose for maintaining fully vaccinated status against COVID-19 and for access to workplaces and other public spaces [10]. The emergence and spread of new or milder COVID-19 variants, such as Omicron, which could warrant updates to currently available vaccines, has fuelled concerns and doubts about the need or effectiveness of COVID-19 vaccination and/or booster shots in both vaccinated and unvaccinated individuals. The public response to the reception of COVID-19 booster vaccines before the mandates contrasted to the epidemiological data collected between June and July 2021 (i.e., 6 months into the national launch of the COVID-19 primary vaccination program), which indicated low rates of COVID-19 vaccine hesitancy for the primary series in the Singapore population (9.9%) [11]. Investigations into the acceptability of COVID-19 vaccines in Singapore reported a high acceptance rate (>80%) [12], and incidences of adverse events (AE) concerning COVID-19 vaccines in Singapore were also consistently low (0.12% as of February 2022) [13].

While vaccine hesitancy has been extensively explored before the COVID-19 pandemic, research on booster vaccine hesitancy, particularly for COVID-19, is limited. With accounts of potentially new COVID-19 variants requiring the adaptation of existing vaccines and repeated doses [14], promulgation of anti-vaccination beliefs, conspiracies and misinformation on social media [15], as well as reports of the decreased severity of Omicron and waning vaccine immunity [16], hesitancy involving booster doses warrants investigation. While the development and availability of primary COVID-19 vaccines were eagerly anticipated in the early stages of the pandemic, with a more stable epidemiological profile and natural immunity conferred by infection rates, hesitancy and reluctance to undergo repeated COVID-19 booster vaccinations may become a challenge in maintaining sufficient immunization coverage against COVID-19.

Research on COVID-19 booster vaccines has been slowly gaining traction. Previous studies have indicated that the rates of COVID-19 booster vaccine hesitancy varied, ranging between 24.7% to 29.0% in several European settings [17,18,19], 24.7% in Jordan [20], 23.4% in Algeria [21], and 6.3% in China [22]. These rates, while diverse, are disconcerting, as they are noted following the completion of the primary vaccination series, which suggests a new onset of hesitancy and/or resurfacing of past concerns. Investigations into people’s beliefs and attitudes on their decision to take the COVID-19 booster vaccine have revealed findings emphasizing the impact of knowledge [23], norms, and perceived control [15] in dictating booster vaccine intentions. Hesitancy to receive or refusal of booster vaccines was also found to be linked with prior experiences or side-effects following the initial COVID-19 vaccination [19], as well as concerns about vaccine safety [18,19] and/or its effectiveness [18], which were aligned with theoretical and empirical evidence from studies on COVID-19 vaccine hesitancy for the primary vaccination series [11,24,25,26]. Reports of adverse events, such as myocarditis [27] and Guillain–Barré Syndrome (GBS) [28], post-vaccination have spurred the World Health Organization and other medical committees to regularly emphasize in their public health communications that the benefits of COVID-19 vaccination outweigh its risks [3,29,30]. While such health messages have been widely used to support COVID-19 vaccine uptake as various vaccination programs were launched (e.g., adult, children, and booster vaccination), it remains unclear how individuals in the community perceive the ratio of benefits versus risks for booster vaccination.

Aside from beliefs and perceptions, negative emotions, such as anxiety and fear, have been linked to COVID-19 vaccine hesitancy for the primary series [31,32], but studies investigating their specific role in hesitancy regarding COVID-19 booster vaccines are scarce. Overall, with the expansion of COVID-19 booster vaccination programmes in several countries and with the gradual relaxation of vaccine mandates and restrictive/containment policies, it is important to better understand the potentially modifiable drivers of COVID-19 booster vaccine hesitancy in order to guide public health efforts aimed at improving COVID-19 booster vaccination confidence and uptake. Hence, the goals of the present study were (a) to determine the prevalence of booster vaccine hesitancy among those who had completed the primary COVID-19 vaccination regimen, and (b) to identify and evaluate the sociodemographic, emotional, and psychological factors associated with COVID-19 booster vaccine hesitancy in Singapore.

## 2. Materials and Methods

### 2.1. Setting and Participants

SOCRATEs (Strengthening Our Community’s Resilience Against Threats from Emerging infections) is a community-based epidemiological cohort proposed to assess the perceptions and knowledge of the Singapore public on infectious disease outbreaks and designed to allow for resurveys between short time periods [33]. Data collection (survey wave 29, cross-sectional study design) was performed on the cohort from 27 October 2021 to 11 November 2021. During the survey period, there were no vaccine or booster mandates and no specified timeline for the completion of COVID-19 booster vaccination following the primary series. However, there were vaccination-differentiated measures in place, which restricted the access of non-vaccinated individuals to some public settings, such as food and beverage establishments and sports activities/facilities. In terms of the COVID-19 epidemiological profile at the end of data collection, Singapore recorded a total of 230,077 cases and 548 deaths [34], with more than 90% of the eligible population (12 years old and above, or 85% of the total Singapore population) having completed their primary COVID-19 vaccination regimen [35].

The SOCRATEs study cohort consisted of participants who were previously recruited from the Health for Life in Singapore cohort study (HELIOS) (Nanyang Technological University, ethics approval IRB-2016-11-030) (*n* = 640), referrals from social media (*n* = 792), and door-to-door visits (*n* = 155). Door-to-door recruitment was conducted by segregating five geographical zones in Singapore, and by randomly selecting an equal number of household units per zone. Referrals and social media were used to facilitate snowball sampling. Eligible participants had to be at least 18 years old and above, either Singaporean or a Permanent Resident, currently living in Singapore, and able to access the survey digitally. When data collection officially commenced, participants received a notification to provide their responses on an online survey platform (FormSG). The survey was conducted in English. No in-person recruitment or contact with participants was made during the entirety of the study period due to heightened COVID-19 restrictions. A total of 1587 participants were recruited, of whom 1552 completed the survey (response rate = 97.8%). The current study was approved by the National Healthcare Group (NHG) Institutional Review Board.

### 2.2. Measures

Sociodemographic information including gender, race, age, occupation status, monthly household income, education, housing, daily regular contact(s), and living arrangements (i.e., living with vulnerable individuals, such as people in poor health, unvaccinated people, the elderly, individuals with immunocompromised states, young children etc., e.g., “Do you live with people who are in poor health?”) were collected via self-report. Anxiety and depression were screened using the generalised anxiety disorder-2 (GAD-2) and patient health questionnaire-2 (PHQ-2) respectively, with total scores of 3 or more suggesting a positive GAD-2 or PHQ-2 result. A validated cut point score of 3, which was found to provide optimal sensitivity and specificity [36,37], was used to classify participants according to probable cases of anxiety and depression [38,39,40].

Guided by prior work on vaccine hesitancy [11,26,31,41] in relation to the primary series of COVID-19 vaccinations, emotions, vaccination cognitions, and threat perceptions were assessed. Participants rated the extent of experiencing emotions such as fear, anxiety, anger, disgust, and helplessness as a result of the COVID-19 pandemic using a single item five-point scale (1 = “Not at all” to 5 = “Extremely”). These items were derived from assessments used in a prior study that evaluated the psychological impact of the COVID-19 pandemic on healthcare workers, patients, and their caregivers in Singapore [41]. Psychological measures included in the survey (Table S1) were developed and reviewed in a prior wave of SOCRATEs [11] and were mapped using constructs derived from theories such as the Health Belief Model [26,42,43,44], the Theory of Planned Behaviour [42,45,46], and Social Cognitive Theory [47,48,49] (Table S2). These included four items regarding COVID-19 risk perceptions (α = 0.76; e.g., “I believe there is a strong likelihood that I would contract COVID-19”), five items on the perceived benefits of COVID-19 vaccination (α = 0.85; e.g., “I believe that COVID-19 booster vaccination is the best measure to get back to a pre-pandemic way of life”), two items on perceived necessity (level of personal need) for booster vaccination (α = 0.78; e.g., “As people in my environment are already vaccinated it is not necessary for me to get the COVID-19 booster vaccination”), and six items on concerns about vaccination (α = 0.89; e.g., “I worry about how the recurrent COVID-19 vaccination booster(s) may affect my health”), which were all assessed on a five-point scale (1 = “Strongly Agree” to 5 = “Strongly Disagree”). Aggregated higher scores signified a higher level for each psychological construct after the appropriate reverse coding was performed.

Scores for all items evaluating perceived benefits and concerns were summed and averaged. Benefits/concerns differential scores were subsequently computed by subtracting the concerns mean scores from the benefits mean scores to obtain a continuous measure, which ranged from −4 to +4, with positive scores indicating more perceived benefits than concerns, and negative scores representing more perceived concerns than benefits for COVID-19 booster vaccines. Although this derived differential score is novel and requires further validation, our approach is guided by relevant theoretical frameworks that highlight the importance of response appraisals for behaviour change, such as the protection motivation theory (threat vs coping appraisals) [50,51], the health belief model (perceived barriers: appraisals of cost versus benefits for a given health action) [44,52,53] (Table S2), and the necessity-concerns framework [54,55], in which the differential scores (operationalised as concerns scores deducted from necessity scores) have consistently been shown to predict medication adherence across a wide range of conditions and patient populations [55].

A single item was used to quantify COVID-19 booster vaccine hesitancy (“When invited [by the health ministry] to take the booster vaccination, would you comply?”), which was prefaced by a short description taken from an online article from 9 October 2021 about the COVID-19 situation in Singapore and a planned rollout of booster vaccines by the Ministry of Health of Singapore (MOH) [56]. Those who reported “Yes” were categorised as non-hesitant: “intent to vaccinate/no booster vaccine hesitancy”, while those who reported “No” and “Unsure” were categorised into the hesitant “booster vaccine hesitant” group.

### 2.3. Statistical Analyses

All analyses were conducted on IBM SPSS 28.0. Frequencies were computed, with mean scores obtained for emotional and psychological constructs. A series of univariate logistic regressions were first performed to identify sociodemographic, emotional, and psychological factors associated with booster vaccine hesitancy. Two double-block multivariable binary logistic regression models combining all parameters were subsequently tested. Sociodemographic variables were entered in the first block using the forced enter method, followed by emotional and psychological parameters (e.g., GAD-2, PHQ-2, helplessness, benefits, necessity etc.) in the second block using a stepwise forward entry method (*p* < 0.05). The first multivariable model included benefits and concerns as separate variables, without the benefits/concerns differential, while the second model included only the differential variable, without the benefits and concerns variables. Continuous variables were standardised to facilitate comparisons of odds ratios. Excluding age, which has the reference category assigned to the oldest group (≥60 years old), the reference category for all categorical variables was designated to the category with the highest frequency. Only participants that had completed their primary COVID-19 vaccination series, but had yet to receive their booster vaccine were included in the analyses. Reliability analyses conducted for all multi-item psychological variables suggested good internal consistency (all α > 0.7; Table S1). The statistical significance level was set at an alpha of 0.05.

## 3. Results

Of the 1552 participants that had completed the survey, 1005 participants (62.1% female, mean age = 42.6 years old, *SD* = 13.5, min/max age = 18–77 years old) met the criteria for the analyses. Missing data (0.2%, *n* = 3), participants who were not fully vaccinated (i.e., less than two doses) (4.8%, *n* = 74), or participants who had already received their COVID-19 booster shot (30.3%, *n* = 470) were excluded. Of the 1005 participants, 69.5% (*n* = 698) indicated their willingness to receive the booster vaccine, while 30.5%, (*n* = 307) reported booster vaccine hesitancy (25.9% (*n* = 260) who were unsure; 4.7% (*n* = 47) who refused the booster vaccine). Of the total, 39.2% (*n* = 394) of respondents reported negative benefits/concerns differential scores. Among those non-hesitant and hesitant about COVID-19 booster vaccines, 24.1% (*n* = 168) and 73.6% (*n* = 226) of respondents reported negative differential scores, respectively (Table 1).

Univariate analyses indicated significant association with the following sociodemographic parameters: age, occupation status, and daily regular contact(s) (Table 1). Emotion variables such as anger, disgust, and helplessness, and psychological factors, such as perceived benefits, necessity, concerns, and benefits/concerns differential were noted as significant standalone correlates of COVID-19 booster vaccine hesitancy. Specifically, respondents who were booster vaccine hesitant reported more anger, disgust, and felt more helpless about the pandemic, had higher concerns about booster vaccines, generally perceived fewer benefits and need for booster vaccination, and were more likely to have lower benefits/concerns differential scores. As indicated by the 95% CIs, fear, anxiety, and COVID-19 risk perceptions were not significantly associated with booster vaccine hesitancy, as the intervals contained the null value (OR = 1) (Table 2).

### Multivariable Models of COVID-19 Booster Vaccine Hesitancy

The multivariable binary logistic regression model was statistically significant, χ^2^(28) = 459.22, *p* < 0.001, had a good fit (Hosmer–Lemeshow χ^2^(8) = 9.427, *p* = 0.308), and accounted for 51.8% (Nagelkerke *R*^2^) of the variance in booster vaccine hesitancy. As per the final step of the multivariable model, education (tertiary education), lower perceptions of booster vaccination benefits, lower perceived need for booster vaccines, higher perceived concerns, and a lower perceived risk of COVID-19 were associated with significantly higher odds of COVID-19 booster vaccine hesitancy. Before any psychological variables were entered, the following sociodemographic factors were significant in the first block of the multivariable model: age, occupation status, and daily regular contact. In the second block (psychological parameters), the following variables emerged as significant multivariable correlates as per the order listed: perceived benefits (step 1), perceived need for booster vaccines (step 2), perceived concerns (step 3), and perceived risk of COVID-19 (step 4) (Table 3). The effects of age, occupation status, and daily regular contact ceased to be significant when these psychological parameters entered the model. None of the emotional variables (i.e., generalized anxiety (GAD-2), depression (PHQ-2), COVID-19-related fear, anxiety, anger, disgust, and helplessness) were statistically significant and did not enter the model at any step.

A second multivariable model, which replaced the perceived benefits and concerns variables with the differential variable, was also significant; χ^2^(27) = 440.55, *p* < 0.001, accounted for 50.1% (Nagelkerke *R*^2^) of the variance in booster vaccine hesitancy and had achieved a good fit (Hosmer–Lemeshow χ^2^(8) = 10.092, *p* = 0.259). The benefits/concerns differential attained statistical significance and entered the model first, followed by the perceived need for booster vaccines, and lastly, COVID-19 risk perception (Table S3). As in the first model, the effect of age, occupation status, and daily regular contact did not remain significant when psychological variables entered the model, and emotion variables did not enter the model, as they were not statistically significant.

## 4. Discussion

The current study evaluated the prevalence and factors associated with COVID-19 booster vaccine hesitancy in the general population of Singapore. Compared to a previous study that determined a low vaccine hesitancy rate of 9.9% for the primary series in Singapore [11], current study findings showed relatively high rates of hesitancy for the booster dose among those already vaccinated. Nearly one-third of respondents (30.5%) reported being booster-hesitant, including 4.7% who said they would refuse the booster vaccine, despite completing the primary COVID-19 vaccine series. From the launch of the booster vaccination programme in September 2021 [5] to the end of the survey (11 November 2021), Singapore recorded a booster uptake rate of 19%, and no booster vaccine mandates were in place during this period [35]. However, MOH subsequently introduced COVID-19 booster vaccination mandates a few months after the conclusion of the study [57], and a 78% COVID-19 booster uptake rate was achieved by June 2022 [58]. While the increase is likely attributed to the introduction of these booster mandates, the observed booster vaccine hesitancy rate before such mandates were implemented highlights a need to improve public confidence about booster vaccination(s) or any recurrent doses, especially as vaccine mandates are lifted in most countries, and offers caution against “public health complacency” due to the success of prior vaccination rollouts.

Univariate analyses indicated that age, occupation status, and daily regular contact were associated with booster vaccine hesitancy. Contrary to prior work that had noted high COVID-19 booster vaccine hesitancy among younger population segments [21,59], our findings showed that the odds for vaccine hesitancy for the booster dose were lower for those between 30 to 44 years and those between 45 to 59 years, compared to respondents in the oldest age category (≥60 years). The higher rate of booster vaccine hesitancy in older respondents in disconcerting, as older individuals are at greater risk of severe outcomes from COVID-19 [60]. While the elderly had been duly prioritised for booster vaccination in the national programme and were invited to take their booster dose during the study window [7], hesitancy among the elderly continued to persist, which led to the launch of vaccine outreach programmes for senior citizens [61]. Hesitancy for booster vaccines was higher among those not employed or in school compared to those who were employed, which was in line with data reported in the USA [62]. Interestingly, the odds of hesitancy were higher for those self-employed than those otherwise employed, which may be explained by vaccination mandates (for the primary series) in work settings. Days before the study commenced, MOH announced a mandate for the completion of the primary series of COVID-19 vaccination for access to workplaces [10]. As self-employed individuals are more likely to be working from home or have more flexibility in choosing their own work environments, they may be less concerned about potential mandates and may have less pressure to comply with vaccination requirements. This may also explain booster vaccine hesitancy among the older respondents. While younger respondents are more likely to be socially and occupationally active and hence, affected by vaccination differentiated safe management measures (e.g., entry to workplaces, dining in at F&B establishments), older individuals may be less concerned or impacted by such measures and restrictions, and thereby could be more undeterred in expressing hesitancy. More work is needed to explore how the various measures taken were perceived and affected decisions related to vaccination among the various population strata. Daily in-person contact with a large number of people (≥50 people) was associated with lower odds of booster vaccine hesitancy. As the risk of COVID-19 exposure increases with the amount of regular contact with other people, an association with vaccine hesitancy was expected. Our finding was consistent with data from China, which found that individuals with higher levels of daily contact (≥21 people) were less hesitant to vaccinate against COVID-19 than those with lower daily contacts (1–10 people) [63]. Education was only significant in the multivariable model after other sociodemographic and psychological concepts were included, which may suggest a possible correlation with other variables. Further investigations are needed to elucidate how education level and its correlation with other parameters may affect intentions and behaviours relating to COVID-19 booster vaccinations.

Study findings indicated that generalised anxiety and depressive symptoms, as screened by the GAD-2 and PHQ-2, respectively, had no significant associations with COVID-19 booster vaccine hesitancy, which were in line with prior studies that employed the same measures to evaluate COVID-19 vaccine intention for the primary series [11,39,40]. However, other studies that had used the full measures (GAD-7 and PHQ-9) to investigate COVID-19 vaccine hesitancy also failed to find significant associations with generalised anxiety and depression [64,65]. This means that measures that consider the context of COVID-19 are needed to assess anxiety and depressive moods. Indeed, a previous study found that anxiety that was assessed using a COVID-19-specific measure (COVID-19 Anxiety Questionnaire [66]) was significantly associated with COVID-19 vaccination willingness, whereas unspecific measures, such as the GAD-2 and PHQ-2, showed no significant associations [39]. Further, the fact that we had assessed emotions using measures that were contextualised within the COVID-19 pandemic and found significant univariate associations for some of these factors further emphasises the need to consider the context when evaluating certain psychological constructs.

As determined in univariate analyses, higher levels of reported anger, disgust, and helplessness in relation to the COVID-19 pandemic were associated with booster vaccine hesitancy. Given that the pandemic has caused a multitude of hardships, such as isolation, social restrictions and financial difficulties, negative responses amounting to frustration or uncertainty could have interfered with motivations in engaging with health behaviours, such as vaccination. [32]. As negative emotions were found to be associated with vaccine attitudes [67], addressing emotions to overcome negative attitudes towards vaccines would be beneficial in reducing hesitancy towards COVID-19 booster vaccination(s) [67,68]. The observed odds ratios in both univariate and multivariable analyses suggested that beliefs about vaccination (i.e., perceived need, benefits, concerns, and cost/benefit evaluations (benefits/concerns differential)) were the main drivers of COVID-19 booster vaccine hesitancy. COVID-19 risk perceptions, albeit significant in the multivariable models after the entry of sociodemographic, emotion and psychological variables, were not reliably associated with booster vaccine hesitancy in univariate analyses. It is likely that the dominance of less virulent COVID-19 variants, such as Omicron, and a high COVID-19 vaccination rate in Singapore could have led to a decrease or more variable perceptions of the threat and risks posed by COVID-19.

Among all other individual psychological parameters, the strongest associations with booster vaccine hesitancy were shown for the perception of benefits. As depicted in the multivariable model (Table 3), a unit increase in perceived benefits indicated a 74.3% decrease in the odds for booster vaccine hesitancy. Additionally, a lower perceived need for COVID-19 booster vaccination was also associated with increased hesitancy. It would thus be imperative to leverage perceptions of personal need, gains, and expected utility in order to bolster booster vaccine uptake. Our findings were consistent with prior work conducted in Singapore, which found a strong association between perceived benefits and need with COVID-19 vaccine hesitancy for the primary series [11]. Given that the COVID-19 pandemic has adversely affected people’s daily lives, the benefits of booster vaccination are likely viewed as vital in fulfilling individual (e.g., reduced likelihood of severe illness) [69] and system-level (e.g., easing restrictions) [69,70] goals that contribute towards attaining pre-pandemic normalcy. Booster vaccination may also be perceived to provide communal benefits, such as preventing COVID-19 transmission to friends and family members, and to protect vulnerable groups, such as the immunocompromised and the elderly.

Enhancing perceptions of the benefits of booster vaccination alone may not be sufficient to support successful vaccination programmes. In tandem with reinforcing the benefits of vaccination, it is also important to continue addressing concerns (if any) about the “maleficence” of vaccination to foster favourable risk-benefit evaluations [18,71]. Several studies have shown that vaccine safety is one of the key factors associated with vaccine hesitancy [69,72,73,74,75,76,77,78]. Issues concerning quality control (e.g., rapid development and approval of COVID-19 vaccines) and side effects were among the most commonly cited safety concerns that have contributed towards COVID-19 vaccine hesitancy [76]. While it is possible that such concerns could dwindle as more progress against the pandemic is made, the novelty of COVID-19 booster vaccines and uncertainties surrounding future booster vaccinations could allow such concerns to persist or even grow. Indeed, the high proportion of individuals with negative benefits/concerns differentials (39.2%) indicates that many do not intuitively endorse common public health messages about “vaccination benefits outweighing the risks”. More than two-thirds (73.6%) of those reporting booster vaccine hesitancy had negative benefits/concerns differential scores. Even among those willing to take the booster vaccine, as many as nearly a quarter (24.1%) perceived more risks than benefits. Investment in sustained public engagement efforts to elicit the perspectives on COVID-19 booster vaccines could thus be crucial in assessing the vaccine literacy of the public and ensuring that their concerns are addressed. Overall, health communication strategies and programmes should focus on allaying concerns surrounding COVID-19 booster vaccines, such as safety, efficacy, and side effects, which could undermine confidence and encourage hesitancy, if left unaddressed. Likewise, the importance of booster vaccination should be emphasized by increasing the perception of personal need and highlighting its benefits for both the individual and the community.

It is important to note that since the conclusion of this study, COVID-19 vaccination policies in Singapore were revised and booster vaccination mandates were introduced. Specifically, reception of the COVID-19 booster dose within 270 days of completing the primary vaccination series is required for all individuals 12 years and above to be considered fully vaccinated and to be exempted from vaccination differentiated measures [57,79]. Even in cases of infection and recovery from COVID-19 after completion of the primary series, receiving the booster dose is required to be considered fully vaccinated. While these measures likely have led to an increased uptake in COVID-19 booster vaccines in Singapore (78% as of 22 June 2022), it is not clear what the uptake of additional COVID-19 booster doses will be if/when such policies are reversed, in line with the relaxation and/or removal of vaccination mandates (e.g., vaccine passes) in other countries such as New Zealand [80], France [81], and Canada [82].

Several study limitations need to be acknowledged. First, as the study adopted a cross-sectional design, no causal inferences can be made. Second, while the study sample was ethnically diverse and represented the ethnic composition of the general population of Singapore, females were overrepresented (national registry = 51.0%, sample = 62.1%), and individuals 60 years old and above were underrepresented (national registry = ~23.5%, sample = 10.9%) [83]. As data collection was conducted using an online survey platform (FormSG), younger participants may have been more likely to respond to the survey, as they tend to have greater exposure to or familiarity with technology. The higher volume of responses from females could be attributed to women having more experiences in medical consultations than men, and would perhaps have greater exposure to health messages concerning vaccination [84]. Third, as the sample was comprised predominantly of individuals of Asian ancestries, it may not be wholly generalizable to non-Asian settings and/or for marginalised communities, without replication. There are also some measurement limitations. To keep the survey brief and facilitate its completion, we have used the GAD-2 and PHQ-2, which was well validated in screening for generalized anxiety and depression [36,37], but was less sensitive than their full measures (cutoff point ≥ 3: GAD-2 = 86%, PHQ-2 = 61%; cutoff point ≥ 10: GAD-7 = 89%, PHQ-9 = 74%) [37,85], and was likely too generic. The use of COVID-19-specific measures, such as the COVID-19 Anxiety Scale [86], is recommended in future research. The study was also limited to assessing COVID-19 booster vaccine intention over its actual uptake. This was because the booster vaccination programme was made available for different age groups in waves, which meant that not all of the adult population strata in Singapore had immediate access to COVID-19 booster vaccines. During the survey period, only adults aged 30 years and above were invited to take the booster [56]. Incorporating COVID-19 booster uptake rate as a behavioural measure should be considered for future studies, when booster shots are made available to all adults. Further, inclusion of measures related to prior experiences with vaccination, attitudes, responses towards COVID-19 policies, and trust, which were not assessed in this study, would be a worthy addition. Although our study was guided by prior empirical research on vaccine hesitancy and included several concepts (perceived risk, benefits, costs, etc.) that were mapped in various theories, it did not test a specific theory, nor was it guided by a single theoretical framework, which restricted its capacity to confidently glean any theoretical predictions related to booster vaccine hesitancy. To further build on our findings, studies grounded in a strong theoretical basis are warranted. Finally, with the emergence of new COVID-19 variants and updates to existing COVID-19 measures and policies, longitudinal studies are needed to evaluate how COVID-19 booster vaccine hesitancy and its associated factors might change over time. Such studies also warrant replication in different settings to determine how vaccine hesitancy is affected by contextual and cultural factors.

## 5. Conclusions

The findings of the current study provide a cautionary note that the success of primary COVID-19 vaccination programmes may not necessarily warrant the uptake of booster and/or recurrent doses of COVID-19 vaccines. Although the majority of participants surveyed were in favour of receiving the COVID-19 booster vaccine, hesitancy has been reported for a substantial number of vaccinated individuals. Even among those willing to receive the booster dose, close to a quarter reported negative benefit vs concerns differential scores. Health communication strategies and measures should aim to maximise the perceived benefits of booster vaccination, while also minimising perceived concerns, and target individual needs for booster vaccination. Additionally, addressing barriers that are more prevalent among certain sociodemographic groups could go a long way towards encouraging and increasing the uptake of COVID-19 booster vaccines.

## Figures and Tables

**Table 1:** Sample characteristics of participants (*N* = 1005) and univariate analyses of sociodemographic, mental distress, and benefits/concerns differential categorical variables as a function of booster vaccine hesitancy.

Variables	Total *N* (%)	Non-Hesitant *N* (%)	Hesitant *N* (%)	*p*-Value	Univariate OR ^1^	95% CI ^2^
**Total**	1005 (100.0)	698 (69.5)	307 (30.5)			
**Gender**						
Male	381 (37.9)	273 (71.7)	108 (28.3)	0.237	0.845	0.639–1.117
Female	624 (62.1)	425 (68.1)	199 (31.9)	Ref		
**Race**						
Chinese	898 (89.4)	619 (68.9)	279 (31.1)	Ref		
Malay	28 (2.8)	22 (78.6)	6 (21.4)	0.281	0.605	0.243–1.509
Indian	53 (5.3)	40 (75.5)	13 (24.5)	0.318	0.721	0.380–1.370
Others	26 (2.6)	17 (65.4)	9 (34.6)	0.701	1.175	0.517–2.667
**Mean Age (SD)**	42.58 (13.5) ^3^	42.3 (13.4) ^3^	43.3 (13.7) ^3^			
**Age**						
18–29 years	210 (20.9)	145 (69.0)	65 (31.0)	0.076	0.648	0.401–1.046
30–44 years	350 (34.8)	254 (72.6)	96 (27.4)	**0.008**	0.546	0.349–0.853
45–59 years	335 (33.3)	234 (69.9)	101 (30.1)	**0.038**	0.623	0.399–0.974
60 years and above ^4^	110 (10.9)	65 (59.1)	45 (40.9)	Ref		
**Highest Education**						
No Formal Education/ Primary Education	21 (2.1)	18 (85.7)	3 (14.3)	0.094	0.349	0.102–1.199
Secondary/ Postsecondary Education	387 (38.5)	276 (71.3)	111 (28.7)	0.227	0.842	0.637–1.113
Tertiary Education	597 (59.4)	404 (67.7)	193 (32.3)	Ref		
**Housing Type**						
1–3 room HDB ^5^	133 (13.2)	90 (67.7)	43 (32.3)	0.560	1.127	0.754–1.684
4–5 room HDB/Executive Apartment/DBSS/HUDC ^6^	628 (62.5)	441 (70.2)	187 (29.8)	Ref		
Condominium/Landed Property	244 (24.3)	167 (68.4)	77 (31.6)	0.608	1.087	0.790–1.497
**Monthly Household Income**						
SGD 4999 and below	307 (30.5)	209 (68.1)	98 (31.9)	Ref		
SGD 5000–8999	283 (28.2)	193 (68.2)	90 (31.8)	0.975	0.995	0.703–1.407
SGD 9000–12,999	230 (22.9)	160 (69.6)	70 (30.4)	0.713	0.933	0.645–1.350
SGD 13,000 and above	185 (18.4)	136 (73.5)	49 (26.5)	0.203	0.768	0.512–1.152
**Occupation Status**						
Employed	603 (60.0)	438 (72.6)	165 (27.4)	Ref		
Student	101 (10.0)	72 (71.3)	29 (28.7)	0.779	1.069	0.670–1.705
Self-employed	133 (13.2)	80 (60.2)	53 (39.8)	**0.005**	1.759	1.190–2.599
Not employed or student	168 (16.7)	108 (64.30	60 (35.7)	**0.036**	1.475	1.026–2.120
**Daily Regular Contact(s)**						
Less than 10 people	500 (49.8)	332 (66.4)	168 (33.6)	Ref		
10–19 people	223 (22.2)	153 (68.6)	70 (31.4)	0.559	0.904	0.645–1.268
20–49 people	187 (18.6)	137 (73.3)	50 (26.7)	0.086	0.721	0.497–1.048
50 or more people	95 (9.5)	76 (80.0)	19 (20.0)	**0.010**	0.494	0.289–0.844
**GAD-2**						
Below cut point (Total score < 3)	897 (89.3)	629 (90.1)	268 (87.3)	Ref		
At or above cut point (Total score ≥ 3)	108 (10.7)	69 (9.9)	39 (12.7)	0.269	1.167	0.887–1.535
**PHQ-2**						
Below cut point (total score < 3)	872 (86.8)	611 (87.5)	261 (85.0)	Ref		
At or above cut point (Total score ≥ 3)	133 (13.2)	87 (12.5)	46 (15.0)	0.595	1.079	0.815–1.429
**Living with people in poor health**						
No	936 (93.1)	655 (70.0)	281 (30.0)	Ref		
Yes	69 (6.9)	43 (62.3)	26 (37.7)	0.184	1.409	0.849–2.339
**Living with unvaccinated** **people**						
No	887 (88.3)	618 (69.7)	269 (30.3)	Ref		
Yes	118 (11.7)	80 (67.8)	38 (32.2)	0.678	1.091	0.723–1.647
**Living with people vulnerable to COVID-19 (e.g., elderly)**						
No	924 (91.9)	645 (69.8)	279 (30.2)	Ref		
Yes	81 (8.1)	53 (65.4)	28 (34.6)	0.413	1.221	0.757–1.972
**Living with children between 0 to 11 years old**						
No	977 (97.2)	682 (69.8)	295 (30.2)	Ref		
Yes	28 (2.8)	16 (57.1)	12 (42.9)	0.156	1.734	0.810–3.711
**Negative Benefits/Concerns** **Differential Score**	394 (39.2)	168 (24.1)	226 (73.6)			
**Positive Benefits/Concerns** **Differential Score**	611 (60.8)	530 (75.9)	81 (26.4)			

*Note:*^1^ Odds Ratio. ^2^ Confidence Interval. ^3^ Mean age, with values in brackets representing standard deviation. ^4^ Reference group is not designated to the category with the highest frequency. ^5^ HDB is an abbreviation for Housing and Development Board, a statutory board that provides public housing in Singapore. ^6^ DBSS/HUDC are a series of premium flats built by private developers, which have since been discontinued.

**Table 2:** Mean scores and standard deviation of all emotional and psychological variables, with univariate analyses performed.

Variables	Total *M* (SD)	Non-Hesitant *M* (SD)	Hesitant *M* (SD)	*p*-Value	Univariate OR ^1^	95% CI ^2^
**Fear**	1.95 (0.94)	1.94 (0.91)	1.99 (1.00)	0.394	1.060	0.928–1.210
**Anxiety**	1.97 (0.97)	1.94 (0.94)	2.04 (1.02)	0.147	1.103	0.966–1.259
**Anger**	1.79 (1.00)	1.74 (0.94)	1.91 (1.13)	**0.020**	1.168	1.025–1.332
**Disgust**	1.66 (0.98)	1.61 (0.92)	1.79 (1.10)	**0.008**	1.191	1.046–1.356
**Helplessness**	1.96 (1.12)	1.91 (1.09)	2.08 (1.20)	**0.026**	1.161	1.018–1.323
**Perceived Risk of COVID-19**	3.65 (0.70)	3.67 (0.68)	3.59 (0.75)	0.116	0.898	0.786–1.027
**Perceived Benefits**	3.52 (0.78)	3.81 (0.61)	2.86 (0.71)	**<0.001**	0.159	0.124–0.205
**Necessity for booster vaccine**	3.35 (0.91)	3.63 (0.83)	2.72 (0.76)	**<0.001**	0.283	0.234–0.343
**Perceived Concerns**	3.19 (0.84)	2.99 (0.81)	3.64 (0.72)	**<0.001**	2.617	2.194–3.122
**Benefits/Concerns Differential**	0.34 (1.41)	0.83 (1.19)	−0.78 (1.22)	**<0.001**	0.187	0.147–0.237

*Note:*^1^ Odds ratio. ^2^ Confidence Interval.

**Table 3:** Final multivariable binary logistic regression model of the variables of COVID-19 booster vaccine hesitancy, with benefits and concerns as separate variables.

Variables		OR ^1^	*p*-Value	95% CI ^2^	
				Lower	Upper
**Sociodemographic Variables**					
**Gender**	Female	Ref			
	Male	0.850	0.405	0.580	1.246
**Race**	Chinese	Ref			
	Malay	0.808	0.707	0.267	2.452
	Indian	0.578	0.208	0.246	1.356
	Others	1.571	0.427	0.515	4.791
**Age**	18 to 29 years	1.110	0.797	0.501	2.460
	30 to 44 years	0.521	0.067	0.260	1.047
	45 to 59 years	0.738	0.359	0.385	1.413
	60 years and above ^3^	Ref			
**Highest Education**	No Formal Education/ Primary Education	0.109	**0.009**	0.021	0.577
	Secondary/Postsecondary Education	0.540	**0.005**	0.352	0.827
	Tertiary Education	Ref			
**Housing Type**	1–3 room HDB	1.180	0.565	0.671	2.075
	4–5 room HDB/Executive Apartment/DBSS/HUDC	Ref			
	Condominium/Landed Property	0.924	0.725	0.595	1.435
**Monthly Household** **Income**	SGD 4999 and below	Ref			
	SGD 5000–8999	1.047	0.852	0.644	1.704
	SGD 9000–12,999	1.241	0.411	0.742	2.077
	SGD 13,000 and above	0.847	0.568	0.479	1.498
**Occupation Status**	Employed	Ref			
	Student	1.666	0.215	0.743	3.733
	Self-Employed	1.618	0.077	0.949	2.760
	Not employed or student	1.013	0.963	0.574	1.788
**Daily Regular Contact(s)**	Less than 10 people	Ref			
	10–19 people	0.837	0.443	0.532	1.318
	20–49 people	0.794	0.383	0.472	1.334
	50 or more people	0.681	0.269	0.344	1.346
**Living with people in poor health**	No	Ref			
	Yes	1.155	0.682	0.580	2.299
**Living with** **unvaccinated people**	No	Ref			
	Yes	1.335	0.317	0.758	2.349
**Living with people** **vulnerable to COVID-19 (e.g., elderly)**	No	Ref			
	Yes	1.100	0.779	0.564	2.147
**Living with children** **between 0 to 11 years old**	No	Ref			
	Yes	1.877	0.235	0.664	5.304
**Psychological Variables (Stepwise Forward Method, in order of entry)**					
**Perceived Benefits**		0.257	**<0.001**	0.193	0.342
**Necessity for booster vaccine**		0.537	**<0.001**	0.421	0.684
**Perceived Concerns**		1.698	**<0.001**	1.314	2.195
**Perceived Risk of COVID-19**		0.790	**0.031**	0.638	0.978

*Note*: ^1^ Odds ratio. ^2^ Confidence Interval. ^3^ Reference group is not designated to the group with the highest frequency.

## Data Availability

The data presented in the study is available on request from the corresponding author.

## Supplementary Materials

The following supporting information can be downloaded at: https://www.mdpi.com/article/10.3390/vaccines10071088/s1; Table S1: Item list for all psychological variables; Table S2: Psychological variables that were linked to theoretical constructs; Table S3: Final multivariable binary logistic regression model of the variables of COVID-19 booster vaccine hesitancy, with the benefits/concerns differential.
