# Supplementary material for: Determining the Prevalence and Correlates of COVID-19 Booster Vaccine Hesitancy in the Singapore Population Following the Completion of the Primary Vaccination Series
Source: Vaccines (Basel). 2022 Jul 6;10(7):1088. doi: 10.3390/vaccines10071088 (PMC9316882; doi:10.3390/vaccines10071088)
Supplement: Supplementary file 1 [file vaccines-10-01088-s001.zip › vaccines-1702744-supplementary.pdf]

Table S1. Item list for all psychological variables.

**Variables**						**Cronbach's Alpha (α)**
Perceived risk of COVID-19	Strongly Agree	Somewhat Agree	Neither Agree nor Disagree	Somewhat Disagree	Strongly Disagree	0.76
▪ I believe there is a strong likelihood I will contract COVID-19.	1	2	3	4	5	
▪ I believe if I were to contract COVID-19 I'd be likely to infect others.						
▪ I believe that if I were to contract COVID-19 it would have serious consequences to my health.						
▪ I believe that my loved ones would have severe consequences to their health if they were to contract COVID-19.						
Benefits of booster vaccination [1]	Strongly Agree	Somewhat Agree	Neither Agree nor Disagree	Somewhat Disagree	Strongly Disagree	0.85
▪ I believe that the COVID-19 booster vaccination is the best measure get back to a pre-pandemic way of life.	1	2	3	4	5	

▪ I believe my likelihood of contracting COVID-19 or suffering significantly from the virus would be lessened if I take the booster vaccination.						
▪ I believe other people will benefit from me being vaccinated including taking the booster.						
▪ Booster vaccines do not benefit my health in general.[2]						
▪ I see no direct personal benefit in taking the booster COVID-19 vaccination.[2]						
Perceived necessity for booster vaccine	Strongly Agree	Somewhat Agree	Neither Agree nor Disagree	Somewhat Disagree	Strongly Disagree	
▪ "I can protect myself against COVID-19 without the COVID-19 booster vaccination with the pandemic in the current state."[2]	1	2	3	4	5	0.78
▪ "As people in my environment are already vaccinated it is not necessary for me to get the COVID-19 booster vaccination."[2]						
Concerns about booster vaccine [1]	Strongly Agree	Somewhat Agree	Neither Agree nor Disagree	Somewhat Disagree	Strongly Disagree	0.89

▪ The haste in the development of COVID-19 vaccines worries me.	1	2	3	4	5
▪ I worry about how the recurrent COVID-19 vaccination booster(s) may affect my health.					
▪ I believe COVID-19 booster vaccines are riskier than the COVID-19 virus itself.					
▪ The risk of side effects and complications worry me.					
▪ I am concerned about long-term safety for COVID-19 booster vaccines.					
▪ I don't believe vaccines in general are safe.					

Note: [1] After the appropriate reverse-coding was performed, the scores of all the items in the 'benefits' variable and 'concerns' variable are added and averaged (lowest possible = 1, highest possible = 5). The mean score for the 'concerns' variable is subtracted from the mean score of the 'benefits' variable, with the highest possible differential score being 4 and the lowest possible differential score being -4. [2] Item is not reverse-coded due to its framing in the given variable.

Table S2. Psychological variables that were linked to theoretical constructs

Theoretical Framework	Description	Variable(s) Linked to Theory
Health Belief Model (HBM)	▪ HBM states that a person's beliefs in a personal health threat along with his/her beliefs in the effectiveness of a given health behaviour or action predicts the likelihood that the behaviour would be adopted.<br><br>▪ The perceived risk of COVID-19 is mapped from the HBM construct of perceived susceptibility, which refers to an individual's perceived likelihood of infection and vulnerability to a given disease.<br><br>▪ The benefits variable was mapped from the HBM construct of perceived benefits which relates to an individual's perception of the effectiveness of a given health behaviour (such as vaccination).<br><br>▪ The concerns variable and differential variable were mapped from the HBM construct of perceived barriers. The construct refers to the obstacles of performing the given health behaviour and is often weighed against its	▪ Benefits<br>▪ Concerns<br>▪ Perceived risk of COVID-19<br>▪ Benefits/Concerns differential

	perceived effectiveness (benefits) to create a cost/benefit analysis (benefits/concerns differential).	
Social Cognitive Theory (SCT)	▪ SCT describes the influence of experiences, the actions of others, and environmental factors on individual health behaviors.<br><br>▪ Expectations is a construct in the SCT which represents an individual's perception of the value or utility of the health behavior in producing an outcome. As expectations are often influenced by prior experiences, negative experiences relating to COVID-19 such as vaccine side-effects or loss of loved ones to COVID-19 could shape people's beliefs and expectations about COVID-19 vaccination.	▪ Benefits<br>▪ Concerns
Theory of Planned Behaviour (TPB)	▪ TPB predicts that attitudes towards a health behaviour, norms/beliefs, and perceived behavioral control with respect to future performance of the target behavior are mediated by intentions.<br><br>▪ In this study, perceived need for booster vaccination is mapped to the constructs of attitudes and beliefs in the theory. The degree to which an individual has a positive or negative evaluation of	▪ Perceived need for booster vaccination

	COVID-19 booster vaccines as well as their personal beliefs about vaccination could inform their level of need and motivation to engage with vaccination.	
Protection Motivation Theory (PMT)	▪ PMT postulates that fear/threat appraisals and coping appraisals induces protection motivations that would either increase or decrease adoption of a given health behaviour in response to the given health threat(s).<br><br>▪ Threat appraisals in the PMT which consist of one's perceived susceptibility and vulnerability to a disease are mapped to assessments of one's perceived risk of COVID-19 infection.<br><br>▪ The concept of evaluating threat appraisals (perceived severity and vulnerability) against coping appraisals (response efficacy and self-efficacy) which collectively dictates behavioural intention (protection motivation) had served as a guide in the producing the benefits/concern differential.	▪ Perceived risk of COVID-19<br>▪ Benefits/Concerns differential

Note: The variables used were mapped from its respective construct within the theoretical framework and does not imply that the variables were testing a specific theory.

Table S3. Final multivariable binary logistic regression model of the variables of COVID-19 booster vaccine hesitancy with the benefits/concerns differential.

**Variables**		**OR [1]**	***p*-Value**	**95% CI [2]**	
				Lower	Upper
	**Sociodemographic variables**				
**Gender**	Female	Ref			
	Male	0.891	0.545	0.612	1.296
**Race**	Chinese	Ref			
	Malay	0.817	0.719	0.271	2.457
	Indian	0.456	0.076	0.191	1.087
	Others	1.822	0.283	0.609	5.455
**Age**	18 to 29 years	1.395	0.402	0.640	3.040
	30 to 44 years	0.588	0.129	0.296	1.167
	45 to 59 years	0.796	0.485	0.420	1.510
	60 years and above [3]	Ref			
**Highest Education**	No Formal Qualifications/ Primary Education	0.100	**0.006**	0.019	0.521
	Secondary/Postsecondary Education	0.559	**0.006**	0.368	0.849
	Tertiary Education	Ref			
**Housing Type**	1–3 room HDB	1.165	0.592	0.667	2.035
	4–5 room HDB/Executive Apartment/DBSS/HUDC	Ref			
	Condominium/Landed Property	0.905	0.652	0.588	1.394
**Monthly Household Income**	S$4999 and below	Ref			
	S$5,000 - $8,999	1.018	0.942	0.630	1.644
	S$9,000 - $12,999	1.185	0.511	0.714	1.965
	S$13,000 and above	0.844	0.555	0.480	1.484
**Occupation Status**	Employed	Ref			
	Schooling	1.701	0.187	0.773	3.742

	Self-Employed	1.647	0.061	0.977	2.777
	Not employed or schooling	1.022	0.939	0.584	1.789
**Daily Regular Contact(s)**	Less than 10 people	Ref			
	10–19 people	0.843	0.456	0.538	1.320
	20–49 people	0.765	0.303	0.460	1.273
	50 or more people	0.683	0.268	0.348	1.341
**Living with people in poor health**	No	Ref			
	Yes	1.230	0.543	0.631	2.397
**Living with unvaccinated people**	No	Ref			
	Yes	1.349	0.289	0.776	2.345
**Living with people vulnerable to COVID-19 (e.g., elderly)**	No	Ref			
	Yes	1.184	0.616	0.613	2.286
**Living with children between 0 to 11 years old**	No	Ref			
	Yes	1.913	0.219	0.680	5.384
**Psychological Variables (Stepwise Forward Method, in order of entry)**					
**Benefits/Concerns Differential**		0.201	**< 0.001**	0.149	0.271
**Necessity for booster vaccine**		0.485	**< 0.001**	0.383	0.615
**Perceived Risk of COVID-19**		0.691	**< 0.001**	0.563	0.847

Note: [1] Odds ratio. [2] Confidence Interval. [3] Reference group is not designated to the group with the highest frequency.
